# Research progress of exosomes in orthopedics

**DOI:** 10.3389/fgene.2022.915141

**Published:** 2022-08-23

**Authors:** Liang Zhang, Yi Lin, Xiannan Zhang, Chen Shan

**Affiliations:** Department of Hand and Foot Surgery, Jilin Provincial People’s Hospital, Changchun, China

**Keywords:** exosomes, bone marrow mesenchymal stem cell, bone remodeling, spinal cord injury, osteoarthritis, bone tumor

## Abstract

Exosomes are nano-extracellular vesicles secreted by a variety of cells. They are composed of a double-layer membrane that can transport a variety of proteins, coding and non-coding genes, and bioactive substances. Exosomes participate in information transmission between cells and regulate processes such as cell proliferation, migration, angiogenesis, and phenotypic transformation. They have broad prospects in the occurrence, development, and treatment of many diseases including orthopedics. Exosomes derived from different types of bone cells such as mesenchymal stem cells, osteoblasts, osteoclasts, and their precursors are recognized to play pivotal roles in bone remodeling processes including osteogenesis, osteoclastogenesis, and angiogenesis. This articlesummarizes the characteristics of exosomes and their research progress in bone remodeling, bone tumors, vascular skeletal muscle injury, spinal cord injury, degenerative disc diseases, cartilage degeneration, osteoarthritis, necrosis of the femoral head, and osteoporosis.

## Introduction

**FIGURE 1:**
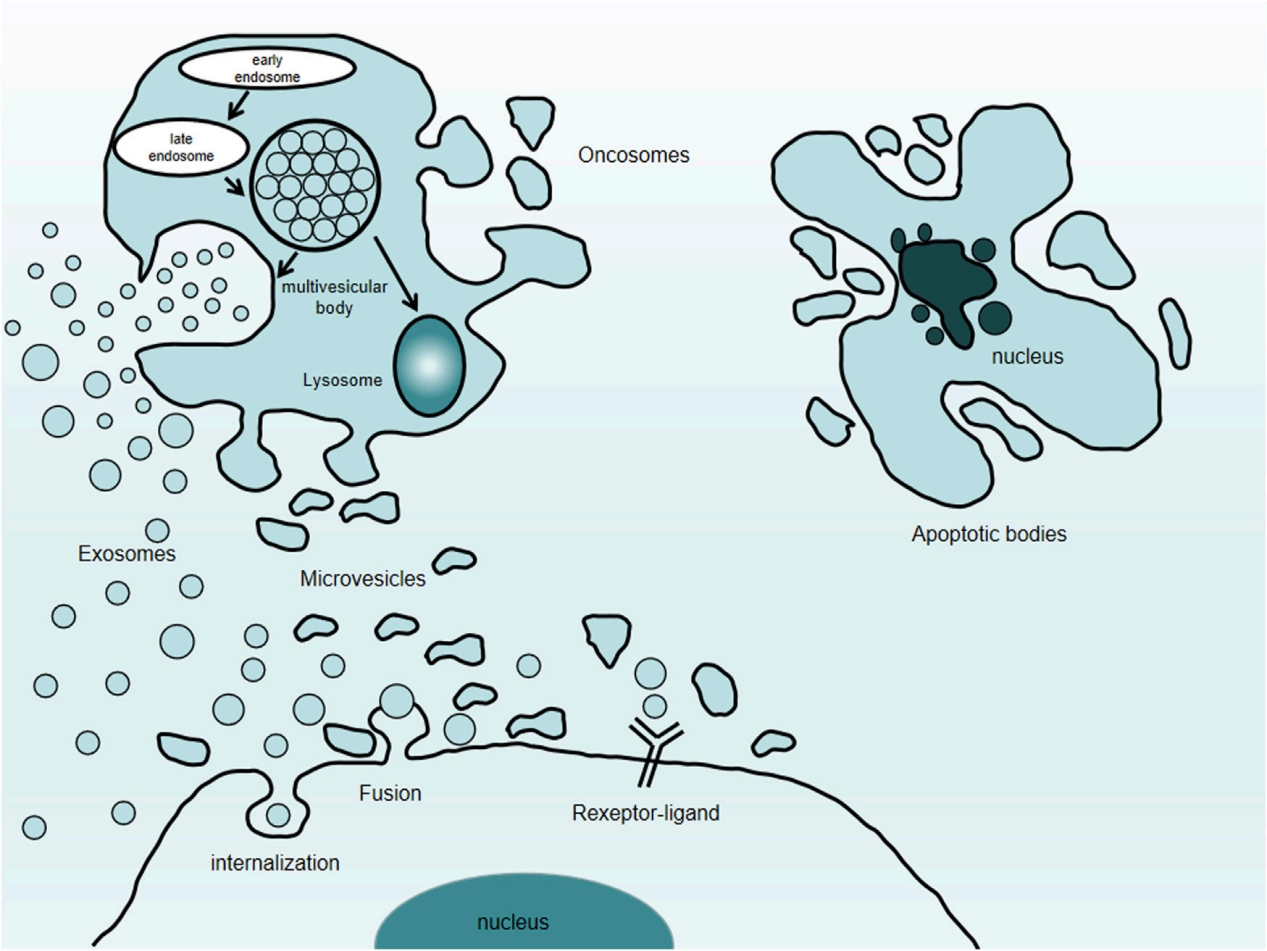
Types of extracellular vesicles. Extracellular vesicles comprise exosomes, microvesicles, oncosomes, and apoptotic bodies.

Exosomes originate from the invagination of the cell membrane and form multivesicular bodies, which then gradually mature into endosomes. After fusion with the cell membrane, exosomes are actively secreted outside the cells (Cruz and Rocco, 2020). The exosome is usually a lipid bilayer vesicle with a diameter of about 30–150 nm (Batsali et al., 2020; Liu et al., 2020). Its double-layered structure can effectively protect the content of the exosomes, including a variety of proteins, nucleic acids, and lipids, thereby participating in the information transmission between cells (Figure 1). It further regulates cell proliferation (Rezaie, et al., 2019), migration, autophagy (Hassanpour et al., 2020), neovascularization, and phenotypic transformation (He et al., 2018). The type of cells and the environment determines the composition and secretion of exosomes (Table 1). Under stress conditions or stimuli, the secretion of exosomes may rise sharply, and the bioactive substances will vary based on the type of stress. Studies have highlighted the variation in exosomal content based on environmental cues, which aid in their roles in different target cells (Xunian and Kalluri, 2020).

**TABLE 1:** Exosomes from different sources in orthopedics research.

Exosome source	RNA in exosome	Advantage
Exosomes derived from platelet-rich plasma	Promote osteogenic differentiation	Rich of active growth factors Excellent potential to promote damage repair
Exosomes derived from Synovium mesenchymal stem cells	Promote chondrocyte proliferation and migration	Sufficient sources
		Promote chondrocyte proliferation
		Strong migration ability
Bone marrow mesenchymal stem cells	Regulate immunity and inhibit chondrocyte apoptosis	Sufficient sources
		Promote tissue repair
		Widely used in multidisciplinary research
Induced pluripotent stem cells mesenchymal stem cells	Promote BMSCs proliferation, Promote osteogenic differentiation	Sufficient sources
		Individualize treatment
		Promote tissue repair

## Review of exosomes

Almost all types of alive cells can produce exosomes. There are some differences in the exosomes from different sources in terms of yield, contents, function, and drug loading. For example, mesenchymal stem cells (MSC) exosomes participate in the process of tissue repair and injury and have the potential to act as drug carriers. Macrophage-derived exosomes can affect the tissue microenvironment by regulating immune function. It also has specific surface proteins and tends to accumulate in the cancer cells (Zhang et al., 2020).

The traditional exosome purification involves collecting the cell culture medium for gradient centrifugation. Generally, the supernatant after low-speed centrifugation (300 × *g*) is taken. At this step, the cells and debris are removed, and the supernatant after further centrifugation cycles, discarding cell fragments (2000 *g*) and large vesicles (10,000 × *g*), is collected. This supernatant is subjected to ultracentrifugation (100,000 × *g*) to obtain the exosomes (Zhang J. et al., 2015). For further purification, exosomes are washed in phosphate-buffered saline, subjected to another ultracentrifugation cycle, collected, and stored at −80°C. This method is widely used for the purification of exosomes from various biological samples such as cell culture medium, urine, and cerebrospinal fluid. However, the use of ultracentrifugation is time-consuming, provides a limited number of purification, and has low efficiency, which limits its wide application in exosome purification. In recent years, ultrafiltration, precipitation, immunoassay, and rapid purification kit of exosomes have been gradually introduced. However, they all have low efficiency with impurities in the exosomal preparation, making them difficult to be popularized. At present, there is no consensus on the separation methods of exosomes, and different purification methods lead to changes in exosomal proteins and bioactive substances contained therein (Liu et al., 2021). Exosomes cannot be stored for a long time after purification from a cell culture medium. The protection techniques mainly include cryopreservation, freeze-drying, and spray-drying (Zhang et al., 2020).

The detection of exosomes was mainly through the assessment of marker proteins and the observation of particle size and morphology of exosomes under the transmission electron microscope. These can be used as markers to detect exosomes from different sources. These proteins include membrane transport fusion protein, heat shock protein, CD9, CD63, and CD81. Protein electrophoresis is one of the methods used to confirm whether the substances extracted from extracellular body fluid and culture medium are exosomes. Combined with the observations of the transmission electron microscope, the exosome purification is confirmed when the extracted substances have a double-layer membrane and cup-shaped morphology, with a size of about 30–150 nm. Nanoparticle tracking analysis (NTA) is a type of optical particle tracking approach that can calculate the concentration and size of the exosomes based on the Brownian motion. The velocity of the particles is analyzed through their movement in liquid, and finally, the particle size and concentration are calculated.

## The role of exosomes in bone remodeling

Osteoporosis, rheumatoid arthritis, and osteoarthritis (OA) are the most common and complex age-related bone diseases in the world (Sućur et al., 2014). The imbalance between osteoclast bone resorption and osteoblast bone formation leads to bone remodeling imbalance (Sućur et al., 2014) and bone microstructure changes, resulting in bone loss and increased risk of bone fragility and fracture. Bone remodeling is a continuous process of repairing bone microstructure damage and aging bone tissue (Withrow et al., 2016). In the bone microenvironment, the cells involved in bone remodeling regulate the reconstruction process by secreting cytokines (Behera and Tyagi, 2018). The role of exosomes in bone remodeling has attracted attention in recent years. Studies have shown that they play an important role in the communication between the bone microenvironment and the cells. Exosomes released by bone marrow mesenchymal stem cells (BM-MSCs) are involved in bone remodeling, especially in the differentiation of osteoclasts and osteoblasts by transferring bioactive molecules to target cells (Xie et al., 2017). Studies show that MSC-derived exosome microRNA (miRNA) is differentially expressed during the osteogenic differentiation of human BM-MSCs. Exosomes derived from human BM-MSCs have the ability to promote bone regeneration (Martins et al., 2016). Cui et al. (2016) showed that the exosomes of MC3T3-E1 cells can promote the differentiation of bone marrow stromal cells (BMSCs) into osteoblasts. The exosomes inhibit the expression and increase of AXIN1 levels by affecting the miRNA of recipient cells' β-catenin expression, thus activating the Wnt pathway. Other studies confirmed that the exosomes produced by cyc454 cells pretreated with myostatin can be absorbed by osteoblast MC3T3 cells. Here, they significantly reduce the key regulator of osteoblast differentiation Runx2 and reduce osteoblast differentiation by downregulating the Wnt signal pathway. More importantly, this inhibition is regulated by the expression of miRNA carried by exosomes. It is proposed that myostatin inhibits osteoblast differentiation by inhibiting the secretion of exosomal miRNA derived from osteoblasts (Qin et al., 2017). Furthermore, it was found that exosomes derived from C2C12 myoblasts can enter pre-osteoblast MC3T3-E1 and promote osteogenic differentiation through miRNA and other substances carried by them (Xu et al., 2018). Here, miR-27a-3p is the main component of exosome osteogenesis. It changed the expression of antigen-presenting cells and activated the Wnt pathway in MC3T3-E1 cells. This study provides a new perspective to understand musculoskeletal interaction and puts forward the possibility of myogenic exosomes as an indicator of abnormal bone remodeling. Qin et al. (2016a)found that exosomes secreted by BM-MSCs can stimulate osteoblast differentiation, but have no significant effect on the proliferation of human osteoblasts cultured *in vitro*. In the exosomes derived from BM-MSCs, three highly enriched osteogenic miRNAs, miR-196a, miR-27a, and miR-206, were found, which may be involved in the exosomes derived from stem cells.

Exosomes have the function of secreting and promoting bone regeneration (Wang and Thomsen, 2021). Li et al. (2016) found that the serum exosome miR-214-3p increases significantly in elderly fracture patients. Further research confirmed that miR-214-3p was enriched in exosomes derived from osteoclasts, which can not only inhibit the role of osteoclasts, but also inhibit the formation of osteoblasts. MSCs are widely used in tissue repair and regeneration. However, limited survival after MSC transplantation hinders the use of MSC in tissue repair and regeneration. The exosomes from adipose tissue-derived mesenchymal stem cells can promote the proliferation and differentiation of primitive osteoblasts (Lu Z. et al., 2017). When adipose tissue-derived mesenchymal stem cells are treated with tumor necrosis factor, the content of Wnt-3a in their exosomes increases, which inhibits the signal of Wnt and promotes the expression of osteogenic genes.

In the process of bone regeneration, neovascularization can not only serve as a source of oxygen and nutrition but also provide calcium and phosphate to help mineralization. Although the functions of exosomes during bone angiogenesis remain unclear, the role of exosomes in stimulating angiogenesis of other tissues and organs has been studied. *In vitro* experiments have shown that placental MSC exosomes can promote endothelial cell proliferation, migration, and tubule formation (Salomon et al., 2013). In addition, exosomes also increase endothelial cell migration by transporting functional enzymes (such as nicotinamide adenine dinucleotide oxidase). Different miR-129 (Vrijsen et al., 2010), miR-136 (Sahoo et al., 2011), and miR-17–92 clusters (Salomon et al., 2013) in exosomes have been shown to regulate endothelial cell proliferation and angiogenesis. Exosomes not only showed angiogenesis *in vitro*, but also had positive results *in vivo*. Studies have shown that MSC-derived exosomes have successfully improved angiogenesis in different animal models. For example, caudal vein injection of MSC-derived exosomes reduced myocardial ischemia/reperfusion injury and improved angiogenesis of the ischemic heart (Bian et al., 2014). Exosomes from human umbilical cord MSC can increase blood perfusion of local tissues (Zhang et al., 2012). Additionally, in the study of tissue engineering, researchers incorporated exosomes from Induced pluripotent stem cells into β-tricalcium phosphate scaffolds. These not only have positive effects on MSC migration, proliferation, and differentiation *in vitro*, but also promote bone regeneration by increasing angiogenesis *in vivo* (Qi et al., 2016). Exosome secretion significantly promotes angiogenesis, which stimulates bone growth and regeneration. Further exploration of the role of exosomes in angiogenesis will help to develop new bone regeneration treatments.

## The role of exosomes in bone tumors

Recent studies found that exosomes are related to tumor progression and metastasis (Weidle et al., 2017). miRNA in exosomes (Table 2) is transmitted as a signal molecule to its target cells by changing information to achieve cell-to-cell communication (Rezaie et al., 2021). Therefore, miRNA can be used as both tumor suppressor and tumor promoter. In excess amounts, it will interfere with the normal cell cycle, affect apoptosis, and promote cell proliferation. Tumor cell invasion and angiogenesis are related to tumor formation and progression. Concurrently, exosomes play an important role in bone development. These functions make exosomes a useful tool for the diagnosis and prognosis of bone tumors (biomarker potential). Osteosarcoma is a common malignant bone tumor in adolescents. The increase in miR-18 levels in exosomes of metastatic osteosarcoma promotes the migration and invasion of non-malignant fibroblasts *in vitro*. The secretion of miR-675 in exosomes from tumor serum samples increases and inhibits the expression of c-troponin 1 in receptor cells, which is related to the metastatic phenotype of osteosarcoma patients. Therefore, miR-675 in exosomes can be used as a biomarker to monitor the metastasis of osteosarcoma (Gong et al., 2018).

**TABLE 2:** Function of exosomes miRNA in Orthopedics.

miRNA	Orthopedics
miRNA-1	Promote the growth of cartilage through HDAC4
miRNA-92a	Promote proliferation of cartilage progenitor cells through PI3K
miR-92a-3p	Regulates cartilage development and homeostasis through Wnt5a
miRNA-95-5p	Regulates cartilage development and homeostasis through HDAC2
miRNA-100-5p	Maintains cartilage homeostasis through mTOR
miRNA-135b	Promotes chondrocyte proliferation and cartilage repair through SP1
miR-140-5p	Enhances proliferation and migration of chondrocytes through RALA
miRNA-144	Promote apoptosis of cartilage progenitor cells through MMP13
miRNA-320	Promote the growth of cartilage through mmp13
miRNA-486	Relevant with the severity of osteoarthritis

Most tumors such as breast, lung, and prostate cancer could metastasize to the bone. Xu Z. et al. (2018) found that miR-21 in exosomes derived from lung adenocarcinoma cells may be involved in osteoclast formation. Karlsson et al. (2016) reported that exosomes derived from mouse prostate cancer cell line trap-c1l impair the proliferation and differentiation of osteoclasts by affecting the expression of dendritic cell-specific transmembrane proteins, tartrate-resistant acid phosphatase, cathepsin K, and matrix metalloproteinase-9. The levels of exosomal miR-141-3p were found to increase in the serum of patients with prostate cancer (Ye et al., 2017). After a series of *in vivo* and *in vitro* experiments, it was proposed that prostate cancer can promote bone metastasis by secreting exosomal miR-141-3p to regulate osteoblast activity.

## The role of exosomes in vascular skeletal muscle injury

Orthopedic diseases are often accompanied by vascular skeletal muscle injury. The success of bone remodeling is closely related to vascular regeneration. Many studies have found that vascular and skeletal muscle regeneration is closely related to exosomes (Qin et al., 2016b). In this regard, miR-494 was found to be abundant in exosomes derived from BM-MSCs (Nakamura et al., 2015). miR-494 participates in C2C12 myogenesis and endothelial cell migration and can promote skeletal muscle and vascular regeneration. There are a large number of angiogenic factors (such as vascular endothelial growth factor and interleukin-6) in exosomes derived from BM-MSCs, which contribute to skeletal muscle regeneration (Behera and Tyagi, 2018b). Although the specific mechanism of exosomes and their miRNAs for vascular muscle injury is not clear, the therapeutic potential of exosomes has attracted the attention of many researchers.

## The role of exosomes in spinal cord injury

When the spinal cord is injured under physiological conditions, immune cells cannot pass through the blood–cerebrospinal fluid barrier to the spinal cord. Mechanical force directly destroys the nerve tissue and endothelial cell membrane, where immune cells accumulate and penetrate into the lesion site to promote an immune response. The inflammatory response is the most important pathological process and plays an important role in the prognosis of spinal cord injury. Owing to the high incidence rate, complicated cases, and unclear pathogenesis, the treatment of spinal cord injury is a great challenge. Recently, exosomes derived from human umbilical cord mesenchymal stem cells has been shown to effectively trigger bone marrow-derived macrophages to inhibit inflammatory response by downregulating the inflammatory cytokines (Kang and Guo, 2022). Human umbilical cord mesenchymal stem cell-derived exosomes can also reduce inflammation and promote the healing of spinal cord injury, providing a new perspective and treatment strategy for spinal cord recovery (Kang and Guo, 2022).

## The role of exosomes in the degenerative disc diseases

Lumbar disc degeneration is the main cause of lumbar disc herniation. Serious degenerative diseases can cause lower back and leg pain and even nerve damage, affecting the ability to work and, consequently, the quality of life. The use of BM-MSCs can differentiate and supplement intervertebral disc cells, promote the proliferation of nucleus pulposus cells, reduce apoptosis, resist inflammation, and have a therapeutic effect on the degradation of the intervertebral disc. However, in clinical application, it is limited by the physiological barrier and long-term survival of cells. Exosomes were reported to play an important role in the treatment of lumbar degenerative diseases by stem cells (Lu K. et al., 2017). Compared with BM-MSCs, exosomes are more stable and easier to store and can survive in a harsh environment, thereby providing a promising treatment for lumbar disc degenerative diseases. Studies showed that miR142-3p in BMSC-derived exosomes could reduce interleukin-1β-induced inflammatory cytokine secretion by targeting mixed lineage kinase 3 (Zhu L. et al., 2020). In addition, mounts of miR-532-5p in BMSC-derived exosomes could inhibit inflammation by targeting RASSF5 (Zhu G. et al., 2020). A vitro study showed that exosomes could result in more than a 50% increase in cell proliferation and a decrease in cellular apoptosis in 3D human degenerative disc cell cultures (Hingert et al., 2020).

## The role of exosomes in cartilage degeneration

Degenerative injury of articular cartilage and reactive hyperplasia of subchondral bone led to clinical manifestations such as joint pain, stiffness, and joint swelling. Some miRNAs secreted by exosomes can inhibit chondrocyte inflammation and promote cartilage development, thus inhibiting cartilage degeneration. They have been shown to play pivotal roles in the treatment of arthritis (Sun et al., 2019). In this regard, miR-320c in exosomes could promote the differentiation of BM-MSCs into chondrocytes. Exosomes derived from mouse BM-MSCs were used to stimulate macrophage polarization to develop into an anti-inflammatory phenotype, thereby maintaining chondrocyte homeostasis and protecting the chondrocytes (Ke and Zhu, 2020). Up to now, miRNA-95-5p (Mao et al., 2018a), and miRNA-100-5p (Wu et al., 2019) were also reported to involve in maintaining bone homeostasis during cartilage degeneration. Currently, the most ideal cell source of exosomes for promoting cartilage regeneration remains ambiguous. miR-140 was loaded into exosomes through electroporation, which was more effective in suppressing the progression of cartilage degeneration and in enhancing cartilage regeneration (Liang et al., 2020). miR-135b in exosomes secreted by transforming growth factor-β1-stimulated MSCs could downregulate Sp1 protein expression, leading to better cartilage regeneration in rats with OA (Wang et al., 2018).

## The role of exosomes in osteoarthritis

OA is one of the most common arthritis. Its pathological features include cartilage degeneration, subchondral bone sclerosis, osteophyte formation, synovitis, and ligament calcification. At present, the main clinical treatment of OA includes oral non-steroidal anti-inflammatory drugs; hence, scholars are still looking for new treatment strategies. Previous studies have focused on stem cell therapy, such as intra-articular MSCs, which can prevent cartilage degeneration and delay the process of osteoarthritis. Subsequent studies have inferred that the therapeutic function of stem cells comes from its paracrine effect, including its secreted exosomes and particles. Tao et al. (2017a) found that the secretion from synovial mesenchymal stem cells (SMSC) contains Wnt5a and Wnt5b which can activate the Yes-related protein signal transduction pathway, thereby promoting the proliferation and migration of chondrocytes. However, activation of the Yes-related protein signal transduction pathway by Wnt5a and Wnt5b can inhibit the expression of Sox9 and significantly reduce the secretion of the extracellular matrix of chondrocytes. The modified SMSC-derived exosomes with high expression levels of miR-140-5p can eliminate this deficiency, promote the proliferation and migration of articular chondrocytes without affecting the secretion of the extracellular matrix of chondrocytes, and prevent the occurrence of OA in rats. Wnt5a is an atypical Wnt protein. The development of joints, including cartilage, bone, and joint cavity, is highly dependent on the Wnt signal transduction pathway (Ge et al., 2009). Wnt5a has dual functions: in the early stage of cartilage formation, it can activate chondrocyte proliferation and inhibit chondrocyte differentiation, whereas, in the late stage, it can activate matrix metalloproteinase and reduce cartilage formation and extracellular matrix synthesis in mature chondrocytes. BM-MSC-derived exosomes with high expression levels of miR-92a-3p have been reported to target the 3′-untranslated sequence of *wnt5a* mRNA, inhibit Wnt5a activity, and inhibit cartilage degradation with homeostasis regulating in OA mice (Mao et al., 2018b). In this study, the biological effect of Wnt5a is similar to the results of SMSC-derived exosomes reported by Tao et al. (2017b). The difference between these studies is that the latter inhibits the negative effect of Wnt5a on cartilage degradation through miR-140-5p and retains the protective effect of Wnt5b on cartilage. Human BM-MSC-derived exosomes also contain long-chain noncoding RNA klf3-as1, which can bind to the miR-206 gene as an endogenous competitive RNA inhibitor, affecting the regulation of the miR-206 gene and further improving the expression of G protein–coupled receptor kinase interacting protein-1 (GIT1) (Liu et al., 2018a). Studies have shown that GIT1 can promote chondrocyte proliferation and inhibit chondrocyte apoptosis (Zhang LQ. et al., 2015). Platelet-derived growth factor is shown to promote chondrocyte proliferation and inhibits chondrocyte apoptosis by upregulating GIT1 expression levels (Zhao et al., 2016). Overexpression of miR-206 and knockout of *Git1* can reverse the therapeutic effect of BM-MSC-derived exosomes on OA, indicating that these exosomes regulate miR-206/git-1-mediated chondrocyte proliferation and apoptosis and promote cartilage repair through klf3-as1 (Liu et al., 2018b). Furthermore, studies have compared the therapeutic effects of BM-MSC-derived exosomes and particles on OA. The results show that they have similar functions and can reconstruct chondrocyte homeostasis, inhibit chondrocyte apoptosis, and stimulate macrophages to polarization toward an anti-inflammatory phenotype, thereby inhibiting cartilage degradation and blocking OA in mice (Cosenza et al., 2018).

## The role of exosomes in necrosis of the femoral head

Glucocorticoid-induced femoral head necrosis is a progressive and disabling joint disease, which is often caused by long-term oral steroids. Apoptosis of BMSCs, osteoblasts, and vascular endothelial cells and the increased differentiation of BMSCs into adipocytes and deformity of bone formation play a key role in this pathological process. Therefore, early prevention of BMSC apoptosis, promotion of BMSC proliferation, and prevention of BMSC differentiation into adipocytes may prevent the progression of glucocorticoid-induced femoral head necrosis. Guo et al. (2016) showed that SMSC-derived exosomes post intravenous injection-induced osteonecrosis of the femoral head in the treated rats. The results indicated that the subchondral bone trabeculae of the femoral head in the treatment group were complete and evenly distributed. Only 20% of the rats had very slight changes in osteonecrosis. The thickness, bone volume (tissue volume), and the number of bone trabeculae in the control group were significantly reduced, resulting in obvious osteonecrosis. The cancellous bone of the femoral head was sparse or even disappeared. This suggests that promoting the proliferation and inhibiting the apoptosis of BMSCs induced by glucocorticoids may be the potential mechanism of SMSC-derived exosomes to prevent femoral head necrosis in rats. Many studies applied various types of exosomes, including mutant hypoxia-inducible factor-1α modified BMSC-derived (Li et al., 2017), multifunctional stem cell-derived (Liu et al., 2017), platelet-rich plasma-derived (Tao et al., 2017a), and BMSC-derived exosomes (Fang et al., 2018), to achieve a better outcome in treating glucocorticoid-induced femoral head necrosis. The principle is to: 1) promote the proliferation of BMSC, osteoblasts, and vascular endothelial cells; 2) promote the osteogenic differentiation of BMSC and angiogenic differentiation of endothelial progenitor cells; 3) resist the apoptosis caused by high glucocorticoid; and 4) activate the cellular protein kinase B/Bad/Bcl-2 and phosphatidylinositol 3-kinase/protein kinase B signal transduction pathway. These different exosomes are effectively prevented by promoting angiogenesis and osteogenesis and the progress of glucocorticoid-induced femoral head necrosis.

## The role of exosomes in osteoporosis

As people get older, the osteogenic directional differentiation ability of BMSCs decreases, which is accompanied by the enhancement of adipocyte differentiation tendency. This imbalance can lead to the decline of osteogenic ability and the accumulation of bone marrow adipose tissue, which is considered to be the pathophysiological mechanism of OP (Deng et al., 2021). Studies have shown that BMSCs exosomes can inhibit adipogenic differentiation while promoting osteogenic differentiation and vascularization, which reverses the imbalance between osteogenic and adipogenic conversion (Chen et al., 2017). The Wnt/β- Catenin pathway is an important signal mechanism for inhibiting adipogenic gene expression and restoring osteoblast proliferation and differentiation. Zhao, et al. (2018) co-culture BMSCs exosomes with osteoblasts, and find that the expression of proteins related to the mitogen-activated protein kinase (MAPK) signal pathway increased, and the proportion of the S phase of the cell cycle increased significantly, indicating that the BMSCs exosomes can promote osteoblast proliferation by activating MAPK signal pathway. A recent study showed the differential expression of microRNA in exosomes during osteogenic differentiation in the process of bone metabolism regulation. The expression of miR-218, let-7a, miR-135b, miR-148a, miR-299-5p, miR-219, miR-135b, miR-302b, and miR-299-5p is significantly up-regulated, while the expression of miR-155, miR-320c, miR-885-5p, miR-181a, and miR-221 is significantly down-regulated, revealing that miRNA may be a potential target of OP therapy (Xu et al., 2014). Zhang et al. (2021) showed that miR-935-modified bone marrow mesenchymal stem cells-derived exosomes enhance osteoblast proliferation and differentiation in osteoporotic rats by targeting STAT1, which provides new therapeutic targets for the treatment of osteoporosis. Cui et al. (2021) pointed out that the engineered exosomes BT-Exo-siShn3 could alleviate excessive bone resorption, insufficient bone formation, and inadequate vascularization, which provided a promising therapy for the treatment of osteoporosis.

## Clinical significance of exosomes

**FIGURE 2:**
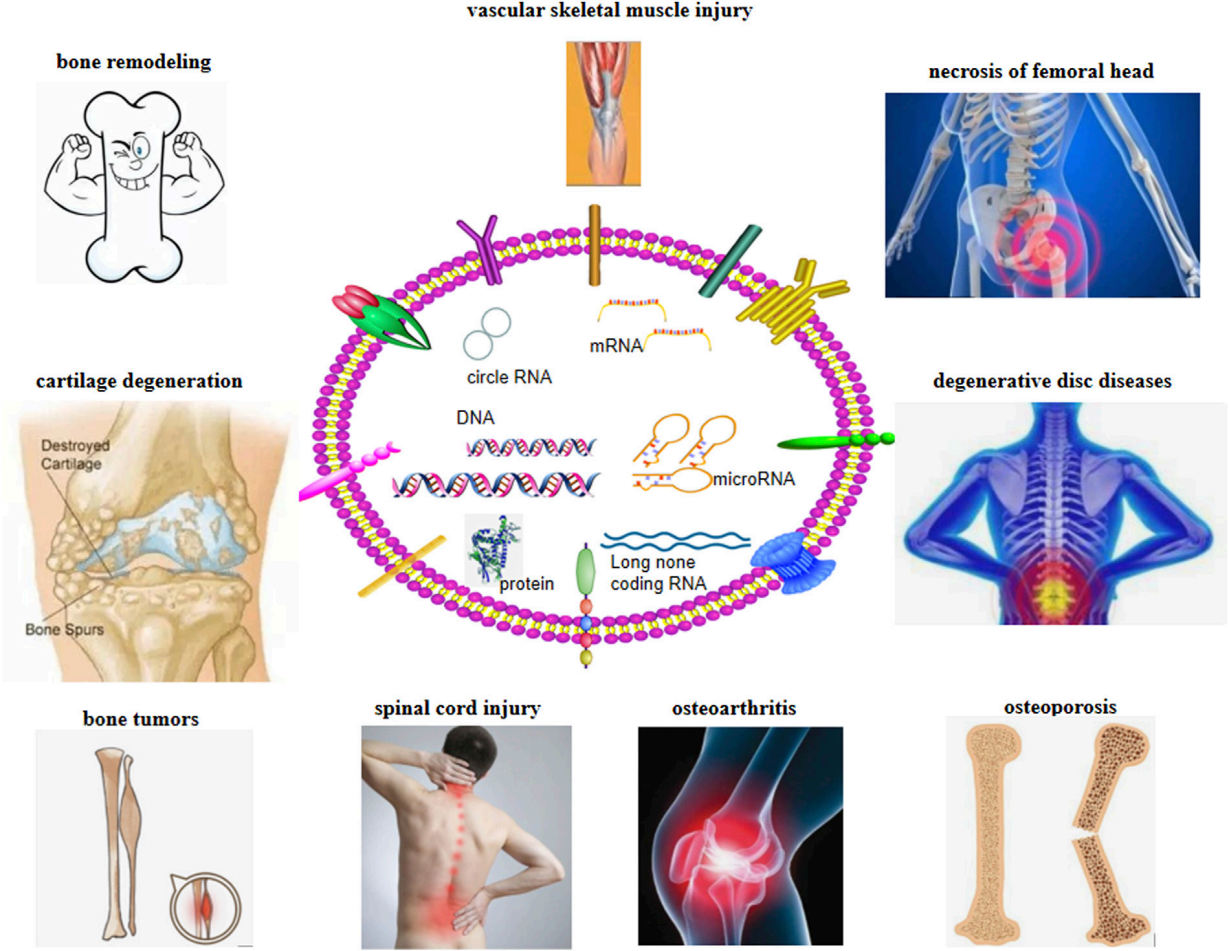
Research progress of exosomes in orthopedics.

Owing to the special biological characteristics of exosomes, an increasing number of people have begun to study their characteristics and treatment potential (Figure 2). The composition of exosomes varies with the type of cells, culture conditions, and even separation methods (Li et al., 2018). Bone remodeling is an important bone repair process in human life (Dimitriou et al., 2011). This complex process requires the coordinated activities of many cells to ensure that the process of bone remodeling (bone formation and resorption) occurs sequentially (Raggatt and Partridge, 2010). If the remodeling process goes out of balance, it will cause bone loss and bone microstructure damage, resulting in increased bone fragility and fracture, and the occurrence of osteoporosis (Robling and Bonewald, 2020). BM-MSCs and exosomes can regulate osteoblasts and osteoclasts, thereby promoting the healing of new bone, neovascularization, and skeletal muscle formation. Due to the nanoparticle size, lipid bilayer structure, and specific antibody on the surface of exosomes, they can also play a role in cell communication and have transmission characteristics, making them ideal candidates for drug delivery systems in the treatment of orthopedic diseases. Compared with the popular lipid nanoparticles, the main advantage of exosomes is that they cause low toxicity and immunogenicity, pass through various physiological barriers, and maintain the stability of their contents due to their lipid (Hu et al., 2021). Considering the bone-promoting ability of BM-MSC-derived exosomes, it is an attractive idea to develop exosomes as targeted delivery materials for molecular therapy of bone diseases. Osteogenic exosomes can be used to carry osteogenic molecules to improve bone diseases such as osteoporosis and delayed or nonunion of fractures (Yu et al., 2021). Concurrently, exosomes and their miRNAs can be used as markers for the diagnosis and prognosis of destructive bone diseases and the monitoring of orthopedic cancers (Bravo Vázquez et al., 2021). BM-MSCs have broad prospects in bone regeneration and various tissue regeneration. However, due to the limited number of donors and invasive collection process, they cannot be implemented widely in the treatment of OA. With the in-depth study of its stem cells, increasing evidence confirms that the role of stem cells depends on the exosomes (Li et al., 2018). Compared to the treatment with BM-MSCs, exosomes have the following advantages in orthopedic treatment: 1) as exosomes do not express major histocompatibility complex on the cell surface, they can overcome the shortcomings of cell transplantation, have higher safety, and reduce the problem of immunogenicity; 2) they are easier to preserve and transport; 3) they are generally not limited by various physiological barriers and have higher efficiency; and 4) lack moral and ethical restrictions, which makes it easier to apply them on a large scale. Exosomes can effectively avoid the phagocytosis of mononuclear macrophages in the human body. It can be widely distributed without activation of opsonin and coagulation factors. However, weak targeting and susceptibility to being quickly cleared in the body are the main disadvantages of exosomes. Surface modification of exosomes is a way to overcome its poor stability and rapid elimination. Treating exosomal surface proteins as anchoring devices or affinity tags can meet the requirements of the particle surface by certain means.

Bone is another frequent location for tumor metastasis (Rezaie et al., 2022). Communication among bone cells, tumor cells, and the bone matrix leads to the remodeling of the homeostatic bone process and induces sclerotic and/or osteolytic injuries. Examples of some miRNAs contained in exosomes in prostate cancer include miR-409 and miR-141 which are upregulated and can lead to bone metastasis (Rajagopal and Harikumar, 2018). In breast cancer, bone metastases have been associated with the presence of miR-10a and miR-10b (Vičić and Belev, 2021).

## Conclusion

At present, the molecular mechanism behind bone remodeling and signal cascade is still unknown. Most exosome research (Table 3) is in the preclinical stage, and the traditional exosome separation and purification methods are not feasible in the clinic. Therefore, one of the biggest challenges in the future is to produce exosomes designed for a wide range of clinical applications, which requires more efficient and accurate qualitative and quantitative acquisition methods of exosomes. Exosomes are more important for the treatment of orthopedic diseases than other gene and cell therapies. Thus, we should pursue research on exosomes, deepen the understanding of exosomes, and find appropriate methods to isolate, purify, and use exosomes to implement them in the diagnosis, monitoring, and treatment of orthopedic diseases.

**TABLE 3:** Exosomes RNA in orthopedics.

Sort	RNA in exosome
mRNA	CD19, BCL2L12, LAMC3, PNPLA5, FGFR4, CHRM2, TMBIM6
miRNA	miR-132, miR-107, miR-149-5p, miR-93-5p, miR-335-5p, miR-4784, miR-106a-5p, miR-145, miR-140, miR-221, miR-381, miR-105, miR-210, miR-29a, miR-488, miR-125b, miR-101 miR-146b, miR-34a, miR-181a, miR-582-5p41, miR-324-5p, miR-21-5p, miR-483-5p, miR-384-5p, miR-155, miR-98, miR-127-5p, miR-16-5p, miR-101, miR-146a
lncRNA	FOXD2-AS1, ANCR, DILC, MIR4435-2HG, SNHG1, SNHG5, HULC, PACER, MEG3, LINC00341, ATB, PMS2L2, MALAT1, ROR, ZFAS1, GACAT3, UFC1, MIAI, DANCR, TM1P3, CTD-2574D22.4, TNFSF10, LOC101928134, CASA2, CHRF, Nespas, H19, THRIL, TUG, P21, CIR, PVT1, XIST, MBNL1-AS1, HOTAIR, FAS-AS1, MSR, PCGEM1
circRNA	circRNA-0000253, circRNA-Rtn4, circRNA-3503, circRNA-103801, circRNA-Ep400, circRNA-0001236, circRNA-0070818, circRNA-004826, circRNA-0071367, circRNA-0003425, circRNA-0014431, circRNA-0070586, circRNA-0061162, circRNA-0007132, circRNA-0024733, circRNA-0082961, circRNA-0063583, circRNA-BRWD1, circRNA-0056285, circRNA-Hmbox1

## Data Availability

The original contributions presented in the study are included in the article/Supplementary Material; further inquiries can be directed to the corresponding author.

## Abbreviations

A, osteoarthritis; mscMSCs, mesenchymal stem cells; abrBM-MSCs, bone marrow mesenchymal stem cells; miRNAs, microRNAs; BMSCs, bone marrow stromal cells; SMSC, synovial mesenchymal stem cells; GIT1, G protein coupled receptor kinase interacting protein-1.
